# Experimental *Neospora caninum* Infection in Pregnant Cattle: Different Outcomes Between Inoculation With Tachyzoites and Oocysts

**DOI:** 10.3389/fvets.2022.911015

**Published:** 2022-05-17

**Authors:** Luís F. Pita Gondim, Milton M. McAllister

**Affiliations:** ^1^Department of Anatomy, Pathology and Veterinary Clinics, School of Veterinary Medicine and Animal Science, Federal University of Bahia, Salvador, Brazil; ^2^School of Animal and Veterinary Sciences, The University of Adelaide, Adelaide, SA, Australia

**Keywords:** neosporosis, bovine, transplacental transmission, abortion, experimental

## Abstract

*Neospora caninum* is a globally distributed abortifacient protozoan of cattle. Experimental infections with *N. caninum* in cattle have provided valuable information on host-parasite interaction and immunopathogenesis. Experimental infection of pregnant cows has been reported in about 20 articles, with most studies using cultured parasite tachyzoites as the inoculum. Only three experimental studies have been conducted in pregnant cows using the parasite's oocysts which are shed by dogs, in large part because transmission experiments using oocysts take more time and are more complex and expensive than experiments using tachyzoites. In this minireview, we discuss differences between *N. caninum* tachyzoites and oocysts as inocula for experimental infection of pregnant cows, as well as the route animals are inoculated.

## Introduction

*Neospora caninum* is a cyst-forming coccidian parasite that was initially observed in dogs with neuromuscular disorders (1, 2) and was named in 1988 after a retrospective work using formalin-fixed paraffin embedded canine tissues (3). In the same year of its classification, *N. caninum* was isolated from dogs and propagated in cell culture (4). Even before the naming of *N. caninum* in 1988, the parasite had been identified by two research groups in calves with encephalomyelitis (5, 6); those authors pointed out that the parasite was distinct from *Toxoplasma gondii* and *Sarcocystis* sp., but they did not name it. Within a few years of its primary description, *N. caninum* was identified as a common cause of bovine abortion worldwide (7–10).

Three natural methods of transmission of *N. caninum* are believed to occur in cattle: (1) ingestion of sporulated oocysts of the parasite (horizontal transmission); (2) transplacental infection (vertical transmission) from a previously infected dam to its offspring (11); or (3) horizontal transmission to a pregnant dam followed by vertical transmission to its fetus (combined horizontal and vertical transmission). Useful technical terms to distinguish these three transmission methods are: (1) horizontal; (2) endogenous transplacental; and (3) exogenous transplacental transmission (12).

Dogs (13) and several other canids (14–16) are definitive hosts of *N. caninum*, shedding oocysts of the parasite in feces after consuming tissues of *N. caninum*-infected mice or cattle in experiments (17). Domestic dogs are the most widely distributed definitive host and are the major source of horizontal transmission to cattle worldwide.

Studies on experimental bovine neosporosis have been conducted using animals inoculated with two stages of *N. caninum*: tachyzoites or oocysts. In the majority of the studies, the inocula consisted of tachyzoites propagated in cell culture. Tachyzoites have been inoculated in cows by different routes, such as intravenous (IV), subcutaneous (SC), intramuscular (IM), intra-uterine, and conjunctival (18–21). Sporulated oocysts were employed as inoculum for pregnant cows in only three studies and the cows were inoculated orally (22–24). This minireview compares the outcome of transplacental infection, abortion or fetal death following experimental administration of *N. caninum* to cattle using tachyzoites or oocysts.

## Experimental Infection in Cattle With *N. caninum* Tachyzoites

In previous studies, prior to identification of the oocyst stage shed by dogs, *N. caninum* tachyzoites were inoculated in cattle and provided valuable data on the immunopathogenesis of bovine neosporosis (19, 25). The rationale for using tachyzoites on experimental infection in pregnant cows is to mimic the propagation of the parasite by two different modes: (1) conversion of bradyzoites to tachyzoites derived from recrudescence of a latent infection; (2) conversion of sporozoites to tachyzoites following ingestion of sporulated oocysts (26). A number of additional studies have inoculated tachyzoites into pregnant cows, and 14 of them are partially summarized in Table 1.

**Table 1:** Experimental infection of pregnant cattle with *Neospora caninum* tachyzoites or tissue cysts (a single report).

**Dose of parasites/ route of infection**	**Number of cows (days of gestation)**	**Parasite strain**	**Rate of vertical transmission/notes**	**Rate of abortion or fetal death/notes**	**Reference**
26 × 10^6^ + unknown number of tissue cysts / IM + SC	3 (129, 126, 81)	NC1, NC2 and NC3	100%	2/2 (100%)/ 1 cow was culled	(18)
3 × 10^6^ / IV + 5 × 10^6^ /IM	2 (120)	BPA-1	100%	NS	(25)
3 × 10^6^ /IV + 5 × 10^6^ /IM	2 (138, 161) 2 (80–95) 2 (115–120)	BPA-1	100% 50% 100%	NS NS NS	(19)
1 × 10^7^ / IV	6 (70) 6 (210)	Nc Liverpool	83.3% 100%	83.3% 0%	(26)
1 × 10^7^ / SC 5 × 10^8^ / SC	8 (140) 6 (140)	NC1	Animals were killed from 14 d.p.i.	Animals were killed from 14 d.p.i.	(27)
5 × 10^8^ / IV 5 × 10^8^ / SC	8 (70) 8 (70)	NC1	100% 50%	100% of fetal death* 50% of fetal death*	(20)
1 × 10^7^/ IV	6 (70) 6 (210)	Nc Liverpool	100% 100%	100% of fetal death* 0% of fetal death*	(28)
1 × 10^7^ / IV	6 (70) 6 (210)	Nc Liverpool	100% 100%	100% of fetal death* 0% of fetal death*	(29)
5 × 10^8^ / IV 5 × 10^8^ / SC	8 (70) 8 (70)	NC1	Animals were killed from 14 d.p.i.	Animals were killed from 14 d.p.i.	(30)
1 × 10^8^ / IV	7 (65) 4 (65)	NC1 Nc-Spain7	100% 100%	100% of fetal death* 50% of fetal death*	(31)
5 × 10^7^ / IV	4 (65) 4 seropositive (IFAT ≥ 1:100)	NC-6 Argentina	75% 67%	Animals were killed at 108 days of gestation	(32)
1 × 10^7^ / IV	6 (70) 6 (70)	Nc-Spain7 Nc-Spain8	100% 100%	100% of fetal death* 100% of fetal death*	(33)
1 × 10^7^ / IV	6 (110)	Nc-Spain7	100% / 1 fetus was not tested	50% of fetal death*	(34)
1 × 10^7^ / IV	6 (110) 6 (110)	Nc-Spain7 Nc-Spain1H	Animals were culled at 10 or 20 d.p.i.	Animals were killed at 10 or 20 d.p.i.	(35)

IV, intravenous; IM, intramuscular; NS, not studied; d.p.i., days post inoculation; * = one or more fetuses were surgically removed from the dams; 02 animals were excluded from the table, as they were inoculated intra uterus (19).

Transplacental infection is consistently achieved by IV injection of *N. caninum* tachyzoites in pregnant cows, even when inoculated in the first trimester and regardless whether the cows are seronegative or seropositive at the time of tachyzoite administration. Intravenous inoculation of high numbers of tachyzoites (10^7^ to 5 × 10^8^) in seronegative cows at 65 or 70 days of gestation induced transplacental infection in almost 100% of animals, and this caused abortion in more than 80% (20, 26, 28, 29, 32, 33, 36). Likewise, IV administration of 5 × 10^7^ tachyzoites to seropositive cows on the 65^th^ day of gestation was associated with transplacental infection in all animals, however that study was terminated after 43 days so it was not possible to definitively conclude whether there would have been an effect on abortion (32).

When pregnant cows are SC inoculated, the spread of tachyzoites is slower in comparison with the IV route, and the innate immune-response of the animals seems to alleviate the rapid propagation of the parasite and the occurrence of fetal lesions (30). A study was performed with cows infected IV or SC at 70 days of gestation with 5 × 10^8^ tachyzoites; 50% of the cows that were SC inoculated presented fetal death, whereas fetal death or abortions were observed in all of the IV inoculated animals (30).

The performance of *N. caninum* strains with high or low-to-moderate virulence (as determined *in vitro* and in mouse bioassays) was evaluated evaluated in pregnant cows (33). The animals were inoculated IV at 70 days of pregnancy with 10^7^ tachyzoites of each strain. Fetal death was observed in all inoculated animals, although it occurred more rapidly in those inoculated with the most virulent strain (33).

In other relevant studies, pregnant cows were inoculated with tachyzoites at 110 or 210 days of gestation. In cows inoculated IV with 10^7^ tachyzoites at 110 days of gestation, transplacental infection also occurred in 100% (*n* = 6) of the tested animals (34); however, abortions were observed in only 50% (3/6), contrasting with the 100% of abortions detected in cows inoculated at 70 days of gestation. In cows inoculated IV with 10^7^ tachyzoites of the NC-Liverpool strain at 210 days of gestation, transplacental infection occurred in all animals, but no fetal death was observed (28, 29); the authors stated that the immunocompetence of the fetus at 210 days plays a critical role in its survival.

Experimental *N. caninum* infection by the intra-uterine route was conducted in a study (19), but is not further considered in this review which focusses on transplacental transmission. In another study, *N. caninum* tachyzoites were inoculated by the conjunctival (2 cows) or IV route (2 cows) with 2.5 × 10^8^ tachyzoites at 161 days of gestation; no transplacental infection occurred in cows inoculated by the conjunctival route, in contrast with those IV inoculated, which had transplacental transmission. In three studies, pregnant cows were inoculated with *N. caninum* tachyzoites using combined IM and IV routes (18, 19, 25); as the IM route was not attempted by itself in any study, outcomes attributable to IM inoculation could not be clearly defined for the purposes of this review.

## Experimental Infection in Cattle With *N. caninum* Oocysts

Bovine abortion outbreaks due to neosporosis continue to be a serious economic problem. Exposure of cattle to oocysts shed by dogs or wild definitive hosts may cause point source exposure of pregnant cows and the occurrence of abortion storms (37–40).

Identification of the domestic dog as a definitive host of *N. caninum* and experimental production of oocysts (13) allowed investigation of exogenous transplacental transmission (i.e., horizontal exposure with vertical transmission) and associated immune responses in cattle infected with *N. caninum* oocysts. The rationale for inoculating oocysts in cattle by the oral route is to simulate ingestion of the parasite in food or water (23).

Experimental infections with *N. caninum* oocysts in cattle were first conducted in calves (41). Oral inoculation of oocysts in seven calves induced infection in all animals, which developed *N. caninum* specific IgG antibodies starting at 2 weeks post inoculation (p.i.). Detectable antibody titers, as well as lymphocyte proliferation response to *N. caninum*, persisted until the moment calves were euthanized, ~2.5 months p.i. (41).

In the first experimental inoculation of *N. caninum* oocysts in pregnant cows, three seronegative cows were orally inoculated with 600 oocysts at 70 days of gestation (22); although infection was confirmed in the inoculated cows, transplacental transmission did not occur. The three cows developed detectable antibody titers starting at 3 weeks p.i. and delivered non-infected calves. In a second study, 19 seronegative pregnant cows were orally inoculated with variable numbers of *N. caninum* oocysts (1,500–115,000) and 17 cows developed IgG antibodies to the parasite (23). Among the 19 inoculated cows, only one received oocysts (70,000) at 70 days of gestation. The remaining cows (*n* = 18) received oocysts at 120–176 days of gestation. After inoculation of oocysts, 10 of 11 cows that became infected and did not transmit infection to their fetuses, had decreasing antibody titers after an apex that occurred between 14 and 42 days p.i. In those cows that presented transplacental infection (*n* = 6), antibody titers continued to increase until delivery of infected calves or at the time of euthanasia. The risk of transplacental infection in the inoculated animals increased with larger doses of oocysts and later than 160 days of gestation. Two of four cows that were infected with 41,000 oocysts at 120–130 days of gestation had *N. caninum* abortion or stillbirth, however death of the stillborn calf was conservatively not attributed to neosporosis, despite having a high antibody titer and typical histological lesions, because it also had an unrelated congenital defect. Seven other cows administered lower doses of oocysts on gestational day 130 did not have transplacental transmission.

A third study was conducted using 18 pregnant cows that received oocysts by the oral route at three different moments of pregnancy (70, 120 or 210 days) (24). Although 40,000 oocysts were administered to each animal, a bioassay indicated that only 127 of the oocysts were viable in each dose (author's note: the shipment of oocysts had prolonged delay of delivery with thawed ice packs). Transplacental infection did not occur in six cows inoculated at 70 days of pregnancy. Abortion was observed in one of the six cows inoculated at 120 days of gestation, while the remaining five cows did not present transplacental infection. Four of six calves from cows administered oocysts at 210 days of gestation had subclinical congenital infections. Seven of the cows that became infected after oocyst inoculation were selected for rebreeding; these seven cows gave birth to non-infected calves in the following pregnancy.

**Figure 1:**
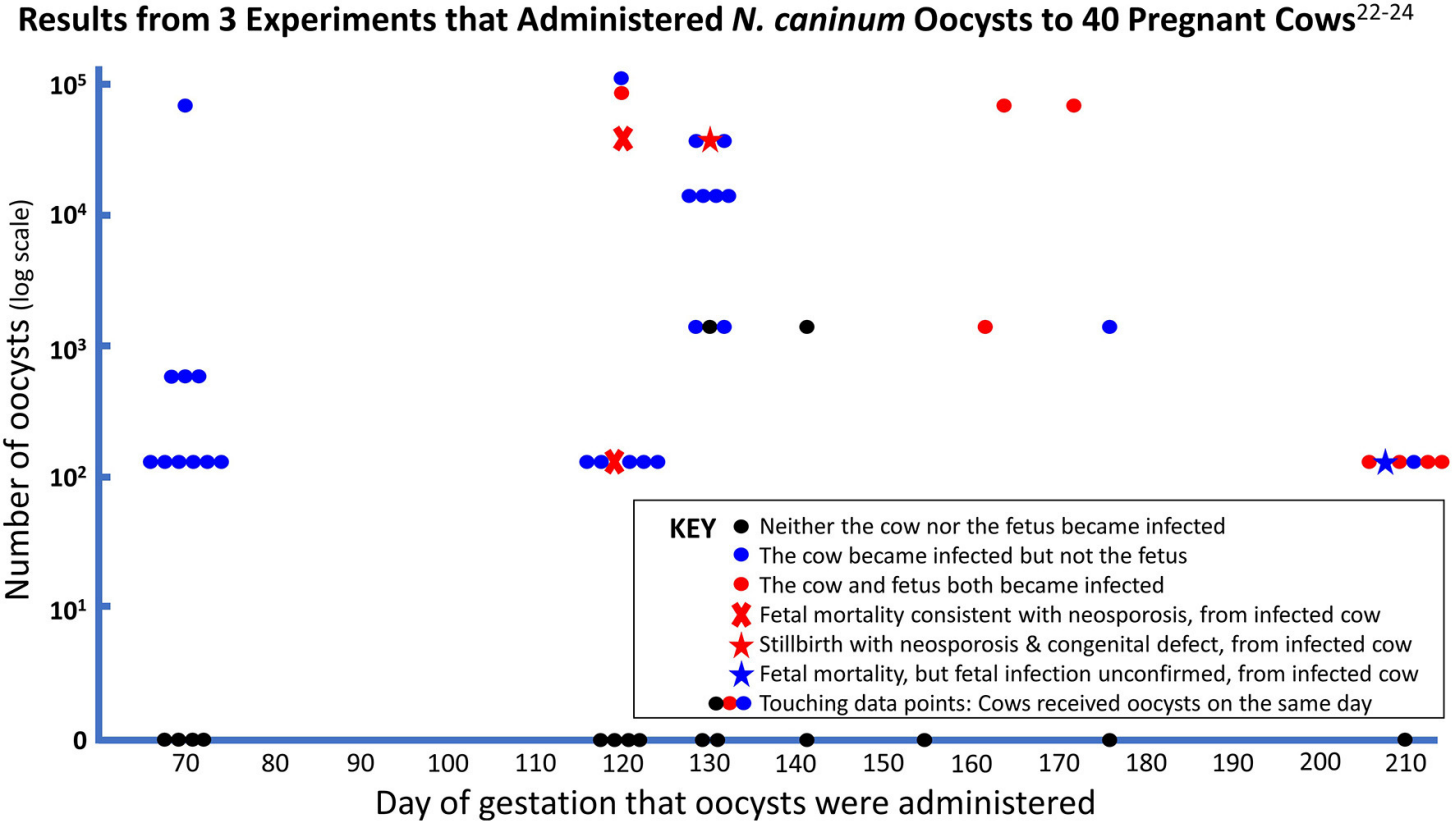
Pregnant cows received between 127 (10^2.1^) and 115,000 (10^5.1^) viable oocysts.

Results of all three studies that used oocysts in pregnant cows are summarized in Figure 1.

## Tachyzoites vs. Oocysts in Experimental Infection of Pregnant Cows

Under natural conditions, it is difficult to precisely predict the outcome of transplacental infection by *N. caninum* in cattle. A single serological screening cannot accurately identify all infected or non-infected cows (31). Experimental infections of pregnant cows with *N. caninum* tachyzoites or oocysts may answer numerous questions, including the outcomes of vertical transmission and fetal lesions or fetal death. Moreover, a bovine model for neosporosis that mimics natural challenge of the parasite is needed to accurately predict the efficacy of potential vaccines.

In the majority of experimental infections in cows, the most frequent times of gestation selected for these studies were 70, 110 and 210 days, which represent the late first, early second, and third trimesters of pregnancy. In most reports, cows were inoculated by the IV route with high numbers of tachyzoites. In only three studies, cows were orally administered *N. caninum* oocysts, and the viability of the oocysts in one of those studies was very low (127 per 40,000), apparently affected by international shipping conditions.

As described above, pregnant cows infected IV at 70 days of gestation with high numbers of *N. caninum* tachyzoites (≥ 10^7^) consistently exhibited transplacental infection and fetal death (20, 26, 28–30). In contrast, in three experiments, no cow orally inoculated at 70 days of gestation with up to 70,000 oocysts transmitted the infection to the developing fetus (22–24). These findings are strong evidence that transplacental transmission of horizontally acquired infection is unlikely to occur in the first trimester of gestation, but when the transmission barrier is overcome by IV inoculation of tachyzoites, then the first trimester fetus is highly susceptible to fatal infection.

Cows infected IV with tachyzoites (≥ 10^7^) at the second trimester of gestation (110 days) consistently presented transplacental infection, but a lower rate of fetal death (about 50%) when compared with cows at 70 days of gestation. When oocysts were administered to 9 cows at 120 days of gestation (127–41,000 oocysts), two neosporosis abortions (22%) were reported among the tested cows (23, 24), as well as a stillbirth with diagnostic complications. Twenty-two percent is well within the range of the abortion incidence of several naturally-occurring neosporosis abortion outbreaks (23), despite six of the nine cows having only received 127 viable oocysts. Abortions occurred 39–44 days after inoculation, corresponding with abortion at about 5$\frac{1}{2}$ months of gestation, which is similar to the average time for naturally occurring neosporosis abortions, reported to be 5.4 months (42).

In the third trimester, both IV inoculation of tachyzoites and oral administration of oocysts led to high rates of transplacental transmission and the birth of congenitally infected but clinically healthy calves. Intravenous inoculation of millions of tachyzoites caused vertical transmission of the parasite in all animals, whereas administration of oocysts later than 160 days, mostly using very low doses (127 viable oocysts), induced subclinical congenital infection in 7 of 10 offspring (70%).

Viewed together, these experiments indicate that transplacental transmission is rare when naïve cows consume oocysts in the first trimester when the fetus is otherwise defenseless, and that transplacental transmission is highly efficient when oocysts are consumed in the third trimester when the fetal immune system is able to withstand the infectious challenge. There appears to be a transitional period, later than 70 days but earlier than 160 days, when transplacental infection occurs in a proportion of pregnancies and is likely to be fatal when it happens, leading to abortion about 6 weeks later. The precision of this observation needs to be improved by experimenting with greater numbers of pregnant cows administered higher doses of *N. caninum* oocysts within this susceptible period. The gestational pattern of neosporosis susceptibility and consequences in cattle appears to be similar to congenital toxoplasmosis in humans (43).

## Future Directions

Despite the difficulty of conducting experimental *N. caninum* infections in cattle, future studies should focus on mimicking, as much as possible, the natural route of infection. Unfortunately, experimental production of *N. caninum* oocysts adds greatly to the complexity, duration, and cost of already expensive bovine gestational experiments. Establishing networks to find, collect, and share *N. caninum* oocysts from naturally infected dogs might provide an alternative method of sourcing oocysts for use in bovine gestational experiments. Due to the sporadic excretion of *N. caninum* oocysts under natural conditions, experimental infection in dogs by feeding them tissues from naturally or experimentally infected animals may be considered; in these bioassays, local strains of the parasite should be used, and the dogs, whenever possible, should be offered for donation after conclusion of the experiment, instead of euthanized, to alleviate ethical concerns of using dogs.

## Author Contributions

LG drafted the manuscript. Both authors contributed to the article and approved the submitted version.

## Funding

LG was recipient of a productivity fellowship by Conselho Nacional de Desenvolvimento Científico e Tecnológico (Process number: 311051/2019-7) from Brazil.

## Conflict of Interest

The authors declare that the research was conducted in the absence of any commercial or financial relationships that could be construed as a potential conflict of interest.

## Publisher's Note

All claims expressed in this article are solely those of the authors and do not necessarily represent those of their affiliated organizations, or those of the publisher, the editors and the reviewers. Any product that may be evaluated in this article, or claim that may be made by its manufacturer, is not guaranteed or endorsed by the publisher.
